# The Impact of Di-2-Ethylhexyl Phthalate on Sperm Fertility

**DOI:** 10.3389/fcell.2020.00426

**Published:** 2020-06-30

**Authors:** Liliya Gabelev Khasin, John Della Rosa, Natalie Petersen, Jacob Moeller, Lance J. Kriegsfeld, Polina V. Lishko

**Affiliations:** ^1^Department of Molecular & Cell Biology, University of California, Berkeley, Berkeley, CA, United States; ^2^Department of Chemistry, University of California, Berkeley, Berkeley, CA, United States; ^3^Graduate Group in Endocrinology, University of California, Berkeley, Berkeley, CA, United States; ^4^Department of Integrative Biology, University of California, Berkeley, Berkeley, CA, United States; ^5^Department of Psychology, University of California, Berkeley, Berkeley, CA, United States; ^6^Helen Wills Neuroscience Institute, University of California, Berkeley, Berkeley, CA, United States

**Keywords:** endocrine-disrupting chemicals (EDC), di-2-ethylhexyl phthalate (DEHP), phthalates, spermatozoa, capacitation, embryo development, acrosome reaction, reactive oxygen species (ROS)

## Abstract

A growing number of studies point to reduced fertility upon chronic exposure to endocrine-disrupting chemicals (EDCs) such as phthalates and plasticizers. These toxins are ubiquitous and are often found in food and beverage containers, medical devices, as well as in common household and personal care items. Animal studies with EDCs, such as phthalates and bisphenol A have shown a dose-dependent decrease in fertility and embryo toxicity upon chronic exposure. However, limited research has been conducted on the acute effects of these EDCs on male fertility. Here we used a murine model to test the acute effects of four ubiquitous environmental toxins: bisphenol A (BPA), di-2-ethylhexyl phthalate (DEHP), diethyl phthalate (DEP), and dimethyl phthalate (DMP) on sperm fertilizing ability and pre-implantation embryo development. The most potent of these toxins, di-2-ethylhexyl phthalate (DEHP), was further evaluated for its effect on sperm ion channel activity, capacitation status, acrosome reaction and generation of reactive oxygen species (ROS). DEHP demonstrated a profound hazardous effect on sperm fertility by producing an altered capacitation profile, impairing the acrosome reaction, and, interestingly, also increasing ROS production. These results indicate that in addition to its known chronic impact on reproductive potential, DEHP also imposes acute and profound damage to spermatozoa, and thus, represents a significant risk to male fertility.

## Introduction

Phthalates and plasticizers are synthetic chemicals that are utilized to make plastic more flexible. They are known to act as endocrine-disrupting chemicals (EDC) (Rudel et al., 2003; Hunt et al., 2009), which are ubiquitous in food and beverage containers, as well as coatings of pills, medical tubing (Green et al., 2005; Hauser and Calafat, 2005) and plastic packaging (Muncke, 2011). Phthalates and plasticizers are bound to plastic polymers by non-covalent bonds, and thus, easily leak into the environment (Pearson and Trissel, 1993). The main routes of exposure to these substances are ingestion, inhalation, dermal absorption, or intravenous medication administration (Hauser and Calafat, 2005; Meeker et al., 2009). Consequently, the vast majority of the population is exposed to these toxins on a daily basis. Low micromolar concentrations of certain EDCs in human urine, sweat and plasma have been associated with an increased rate of miscarriages and compromised male and female fertility (Lovekamp and Davis, 2001; Duty et al., 2003; Hauser et al., 2006; Svechnikova et al., 2007; Burdorf et al., 2011; Toft et al., 2012; Wang et al., 2012; Bloom et al., 2015; Patel et al., 2015; Brehm and Flaws, 2019).

In the present study, we evaluated the reproductive outcomes of acute exposure to four omnipresent EDCs – Bisphenol A (BPA), dimethyl phthalate (DMP), diethyl phthalate (DEP) and Di-ethyl hexyl phthalate (DEHP). BPA, a plasticizer manufactured in large volumes for the production of polycarbonate plastics and epoxy resins, is used to line food and beverage storage containers, coat water supply pipes and is also a component of dental fillings. The aforementioned exposure routes lead to detectable levels of BPA in human serum (Vandenberg et al., 2007), urine (Calafat et al., 2005; Hauser et al., 2007), adipose tissue (Fernandez et al., 2007) and breast milk (Sun et al., 2004). Numerous studies have shown that chronic exposure to BPA alters fertility in both males and females (Zota et al., 2014; Zhou et al., 2017).

Phthalates are similarly produced in large volumes and are used as plasticizing agents. Phthalates can be grouped into two broad categories: low-molecular-weight and high-molecular-weight phthalates. The low-molecular-weight phthalates, such as DEP and DMP, are commonly found in cosmetics and personal care products, respectively. Specifically, DEP is utilized as a solvent and a fixative in fragrances. Studies have shown that chronic exposure to DEP may lead to multigenerational effects on reproductive health in both male and female rats (Fujii et al., 2005). The second most common low-molecular-weight phthalate is DMP. DMP is used primarily as an insect repellent, resulting in extensive exposure due to generous application to exposed skin and clothing. A recent study in mice found that chronic exposure to DMP triggers changes in the levels of serum hormone that lead to increased rates of ovarian granulosa cell death (Mei et al., 2019).

The high-molecular-weight phthalates, such as Di-ethyl hexyl phthalate (DEHP), are used in construction materials and numerous polyvinyl chloride (PVC) products. DEHP is one of the most commonly used phthalates (Wang et al., 2019) and is of primary interest regarding its disrupting impact on fertility. In fact, 98% of the US population test positive for DEHP and its metabolites (Api, 2001; Zota et al., 2016). Despite numerous reports on its toxicity, DEHP is still widely used in consumer products and in a number of medical devices, such as blood bags, infusion tubes, nasogastric tubes, peritoneal dialysis bags, and urological catheters. Patients who undergo frequent hemodialysis, catheterization or massive blood transfusions are at particular risk for DEHP toxicity and are exposed to doses as high as 168 mg/day (Kavlock et al., 2002). Several human studies have demonstrated the profound effects of prolonged exposure to DEHP on both male and female fertility (Burdorf et al., 2011; Pan et al., 2011; Sumner et al., 2019).

The majority of studies on EDC’s impact on reproductive health, including those mentioned above, evaluated the toxic effects of chronic exposure to phthalates and plasticizers. However, little is known about the reproductive outcomes of short exposure to such EDCs.

In the present study, we assessed the effects of acute exposure to BPA, DMP, DEP, and DEHP. Out of the four EDCs tested, DEHP demonstrated the strongest effect on male fertility by significantly altering the maturation process sperm undergoes, also known as capacitation, as well as inhibiting acrosome reaction, and triggering excessive reactive oxygen species (ROS) production. Altogether these changes led to sperm inability to fertilize eggs. These results suggest that DEHP can directly affect sperm fertility and is therefore detrimental to male reproductive health.

## Results

### Murine Embryo Development Is Impacted by DMP, BPA, DEP, and DEHP

**FIGURE 1:**
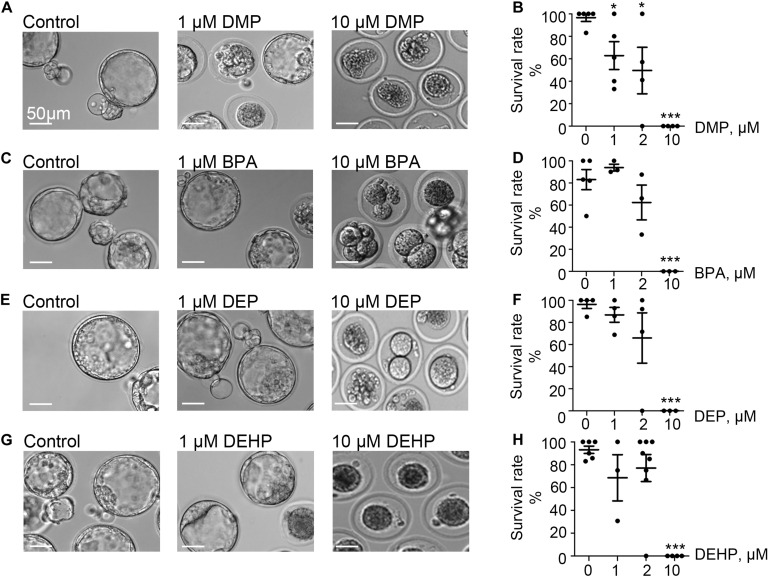
Murine embryo development is impacted by DMP, BPA, DEP and DEHP. *In vitro* embryo development on day 5 post fertilization. Panels **(A,C,E,G)** are representative images of blastocysts previously exposed at the zygote stage to 0, 1, or 10 μM of the indicated EDC for 20 h. The subsequent embryo culture was done in the absences of the indicated EDC. **(A)** Shown are representative images of DMP-exposed embryos. **(B)** The survival rate of DMP exposed zygotes was calculated based on the percentage of embryos that have reached the morula or blastocyst stage. **(C)** Representative images of embryos previously exposed to BPA. **(D)** The survival rate of BPA exposed zygotes was calculated as in **(B)**. **(E)** Representative images of embryos previously exposed to DEP. **(F)** The survival rate of DEP exposed zygotes was calculated as in **(B)**. **(G)** Representative images of embryos previously exposed to DEHP. **(H)** The survival rate of DEHP- exposed zygotes was calculated as in **(B)**. Data are means ± S.E.M. Asterisk indicates a statistical difference between control embryos and embryos exposed to EDCs. *P ≤ 0.05, **P < 0.01, ***P < 0.001. Scale bars for all images are 50 μm.

Exposure to phthalates could either damage sperm directly or impair pre-implantation embryo development after fertilization occurs. To test the susceptibility of pre-implanted embryos to DEHP, DMP, DEP, and BPA, naturally derived zygotes were harvested and subjected to 0, 1, 2, and 10 μM of each EDC as outlined in the methods section. The ability of the pre-implantation embryo to progress toward the blastocyst stage was recorded on day 5 post-fertilization (Figure 1 and Supplementary Figure S1), and the respective survival rate was calculated as described in methods. While all four tested compounds did not affect pre-implantation development at the lower concentrations (up to 2 μM), we found that at 10 μM, all four chemicals effectively prevented blastocyst formation (*p* < 0.05; Figures 1A–H and Supplementary Tables S1A–D). All controls have been performed with either vehicle control (0.1% ethanol) or EDC-free media. No significant differences were observed among control conditions (Supplementary Table S2).

### *In vitro* Fertilization Is Affected by DEHP

**FIGURE 2:**
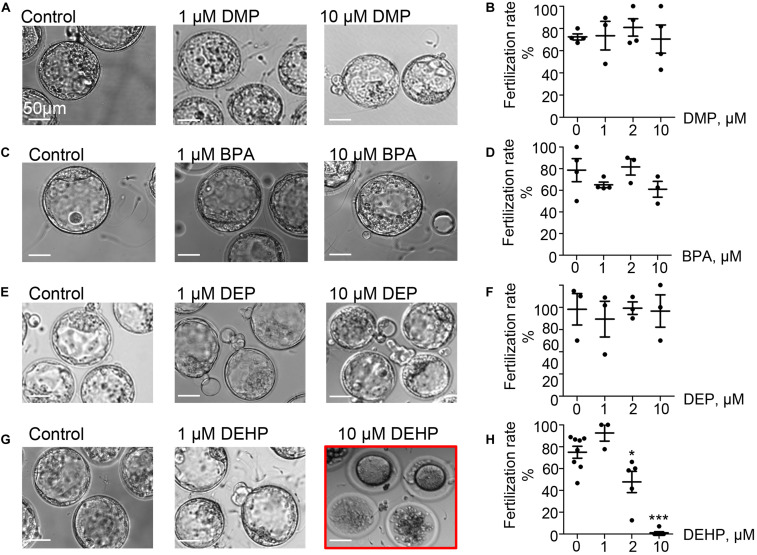
Fertilization rate of murine eggs exposed to EDCs treated spermatozoa. Panels **(A,C,E,G)** are representative images of blastocysts obtained after murine eggs were introduced to sperm previously exposed to 0, 1, or 10 μM of the indicated EDC. Subsequent embryo culturing was done in the absences of EDCs, and the images were taken on day 5 post insemination. **(A)** Shown are representative images of blastocysts obtained after murine eggs were introduced to sperm previously exposed to DMP. **(B)** Percentage of eggs that were fertilized by DMP- treated sperm and were able to reach morula or blastocyst stage. **(C)** Representative images of embryos obtained after IVF with BPA-treated spermatozoa. **(D)** The percentage of eggs fertilized by BPA-treated sperm was calculated as in **(B)**. **(E)** Representative images of embryos obtained after IVF with DEP-treated sperm. **(F)** The percentage of eggs fertilized by DEP-treated sperm was calculated as in **(B)**. **(G)** Representative images of embryos obtained after IVF with DEHP-treated sperm. **(H)** The percentage of eggs fertilized by DEHP-treated sperm was calculated as in **(B)**. Data are means ± S.E.M. Asterisk indicates a statistical difference between control embryos and embryos exposed to EDCs. *P < 0.05, **P < 0.01, ***P < 0.001. Scale bars for all images are 50 μm.

To explore the direct effect of DEHP, DMP, DEP, and BPA on sperm fertilizing ability, *in vitro* fertilization (IVF) assays were carried out. Mouse sperm were capacitated in phthalate-supplemented media as described in methods by exposing sperm to different concentrations (0, 1, 2, and 10 μM) of EDCs, and 60–90 min post-exposure, sperm was introduced to healthy murine eggs. The fertilization rate was calculated and presented as the percentage of embryos that reached the morula or blastula stage on day 5 post-fertilization (*p* < 0.05; Figures 2A–H, Supplementary Figure S2, and Supplementary Tables S3A–D). As shown in Figure 2, all tested concentrations of DEP and DMP did not produce any effect on sperm fertilizing ability and subsequent blastocyst formation, while 10 μM BPA had a minimal, but not a statistically significant impact on early embryo development (Figures 2C,D and Supplementary Figure S2). The most damaging effect to blastocyst formation was observed with DEHP (Figures 2G,H). While spermatozoa retained their fertilization potential at 1 μM, a significant decrease in embryo progression to blastulae was found already at 2 μM (74.95 ± 5.459% in control vs. 47.68 ± 9.68% in 2 μM). Moreover, at 10 μM of DEHP, almost no blastocyst formation was observed (Figures 2G,H). All controls have been done with either 0.1% ethanol as a vehicle control or EDC-free media, and no significant differences were detected between the control conditions (Supplementary Table S4).

### DEHP Prevents Fertilization

**FIGURE 3:**
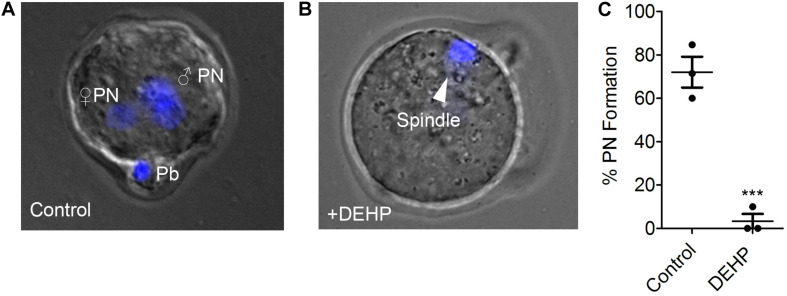
Pronuclei formation after *in vitro* fertilization by DEHP-treated sperm. Pronuclear formation was assessed 9 h after IVF; genomic DNA staining was done with DAPI. **(A)** Representative image of a successfully fertilized egg with two pronuclei (PN) and a polar body (Pb). The egg was inseminated with sperm treated with vehicle control solution. **(B)** Shown are representative images of unfertilized eggs following an IVF with sperm that was treated with 10 μM DEHP. The arrowhead indicates the position of the second metaphase spindle. **(C)** Percentage of fertilized eggs with PN detected. Each data point represents the mean of one of the three independent experiments ± S.E.M. *** indicates statistical significance (*P* < 0.005). The total number of eggs: 25 (control), 36 (+10 μM DEHP).

The acute exposure to DEHP may either affect sperm ability to fertilize the egg or may permit fertilization but subsequently inhibit the zygotic division. To distinguish between these two scenarios, sperm fertility was assessed by recording pronuclei formation 9 h post IVF. As shown in Figure 3, in the presence of 10 μM DEHP, a 92.76% reduction in pronuclei formation was detected compared to untreated control. Values were calculated based on pooled data from three independent experiments and represent a total of 19 zygotes out of 25 eggs in the control conditions, versus 2 zygotes out of 36 eggs in the presence of DEHP (Supplementary Table S5). These results indicate that even short exposure to DEHP modifies sperm physiology making spermatozoa unable to penetrate the zona pellucida.

### Murine CatSper Is Not Affected by DEHP

Since DEHP has demonstrated the most substantial effect on sperm fertility among all tested EDCs, we further explored which sperm functions were directly affected by exposure to this phthalate. Once deposited inside the female reproductive tract, mammalian spermatozoa must undergo a final maturation step, i.e., capacitation, to become competent to fertilize the egg (Austin, 1951; Yanagimachi, 1994). This process results in the removal of non-covalently attached glycoproteins, depletion of cholesterol, and other steroids (Davis, 1981), as well as the removal of adherent seminal plasma proteins (Chang, 1957). These physiological changes alter sperm membrane potential and make the cell competent to undergo a change in motility, known as hyperactivation, trigger the acrosome reaction and prepare spermatozoa for fertilization. Hyperactivation is characterized by calcium influx into the sperm flagellum via the calcium channel- CatSper (Ren et al., 2001; Carlson et al., 2003) and is defined as an asymmetrical flagellar beat that is required for penetration through the viscous luminal fluids of the female reproductive tract and the protective vestments of the egg. CatSper deficiency, as well as its suppression by environmental toxins, has been previously linked to male infertility (Ren et al., 2001; Qi et al., 2007; Schiffer et al., 2014; Tamburrino et al., 2014). To investigate whether murine CatSper is also affected by DEHP, we used murine sperm patch-clamp technique (Kirichok et al., 2006). As shown in Supplementary Figures S3A,B, the application of 10 μM DEHP did not alter CatSper currents. Since previous studies on several EDCs reported that phthalates impact human CatSper at micromolar concentrations (Schiffer et al., 2014), we also tested DEHP at a higher dose (Supplementary Figures S3A,B). However, at either 10 or 100 μM concentration, no significant changes in monovalent CatSper currents were observed. This indicates that DEHP affects sperm cells through a CatSper-independent mechanism.

### DEHP Alters Sperm Capacitation and ROS Production

**FIGURE 4:**
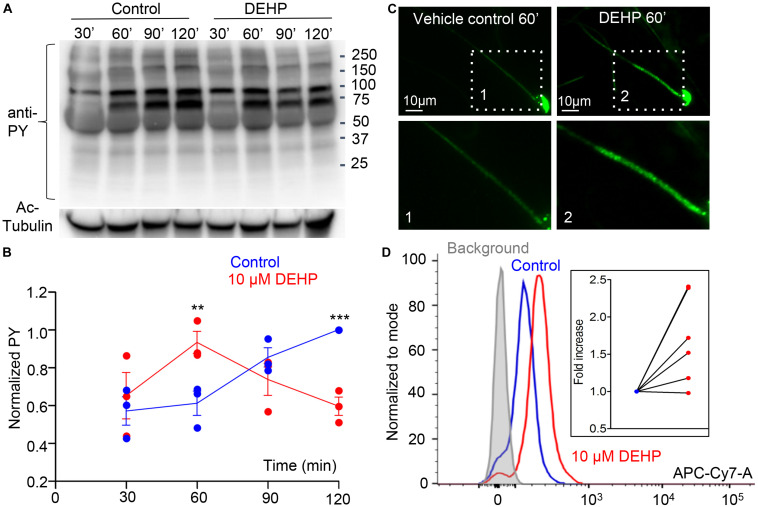
Capacitation-associated tyrosine phosphorylation of murine sperm is altered in the presence of DEHP. **(A)** Representative western blot image shows the time course of protein tyrosine phosphorylation under capacitating conditions in the presence or absence of 10 μM DEHP. DEHP was added to the media immediately before the start of the capacitation process. Sperm lysates were obtained at the indicated times (30, 60, 90, and 120 min) and subjected to SDS-PAGE immunoblotting. Tyrosine phosphorylation was detected with a monoclonal phospho-tyrosine (PY) antibody. Acetylated-tubulin (Ac-Tubulin) was used as a loading control. **(B)** Levels of relative tyrosine phosphorylation obtained as total densities extracted from **(A)** and normalized to the densities of the loading control. Each data point represents the mean of one of the three independent experiments. **(C)** Immunofluorescent localization of tyrosine phosphorylated proteins as visualized by PY antibody. Increased phosphorylation detected after 60 min of capacitation in the mid-piece region of spermatozoa in DEHP- treatment group (right panels) as compared to control untreated spermatozoa (left panels). Lower panels represent insets from the corresponding region of interests indicated on the upper panels by dashed rectangular. **(D)** A representative flow cytometry data showing an increase in global tyrosine phosphorylation in 10 μM DEHP- treated spermatozoa (red) at 60 min of capacitation compared to the vehicle control (blue). Tyrosine phosphoproteins were detected using a CF 647 dye conjugated to an anti-PY antibody. Inset: fold increase in mean fluorescent intensity normalized to mode as detected by the flow cytometer compared to control conditions. Data are means ± S.E.M. ** indicates statistical significance (*P* < 0.01) between control spermatozoa and spermatozoa exposed to 10 μM DEHP. *** indicates statistical significance (*P* < 0.001).

Another hallmark of capacitation is the phosphorylation of sperm proteins on tyrosine residues (Salicioni et al., 2007; Visconti, 2009). Previous reports on the capacitation of murine sperm demonstrate a time-dependent increase in the phosphorylation of tyrosine residues in proteins with the molecular weight of 40—170 kDa (Visconti et al., 1995a; Naz and Rajesh, 2004; Sepideh et al., 2009; Visconti, 2009). This modification allows sperm to hyperactivate, undergo the acrosome reaction and interact with the zona pellucida (Nassar et al., 1999; Naz and Rajesh, 2004). To test the effect of DEHP on sperm tyrosine phosphorylation, caudal epididymal spermatozoa were incubated in a capacitating medium containing either 10 μM DEHP or vehicle control. Subsequently, tyrosine phosphorylation was assessed by a western blot using a monoclonal anti-phosphotyrosine antibody (anti-PY) (EMD Millipore). As shown in Figures 4A,B, DEHP markedly alters sperm capacitation-associated tyrosine phosphorylation kinetics, by expediting the process within the first 60 min of exposure, followed by a complete reversal after 120 min of incubation. Specifically, at the 60-min time point, a 150% increase in the global tyrosine phosphorylation was detected in DEHP treated spermatozoa in comparison to the control condition. As capacitation progressed, the detected levels of the global tyrosine phosphorylation increased in the control samples. However, in the DEHP treated samples, the levels of detected phosphotyrosine significantly dropped. Specifically, at the 120-min time point, the detected levels of phosphoproteins were 59% lower in the control. The most striking changes in phosphorylation were observed at the regions that correspond to 75, 95, 170, and 270 kDa (Supplementary Figure S4). The 75, 95, and 170 kDa molecular weight proteins were previously reported to have important roles in sperm fertility (Naz and Rajesh, 2004; Sepideh et al., 2009). In addition, immunocytochemistry experiments using the same anti-PY antibody, revealed that the increased protein phosphorylation caused by DEHP is primarily localized to the mid-piece region of sperm (Figure 4C). According to previous reports, during the normal capacitation process, the sperm midpiece is undergoing more robust tyrosine phosphorylation in comparison to other parts of the flagellum (Alvau et al., 2016), and it appears that DEHP exacerbates this process. To further assess the global changes in sperm tyrosine phosphorylation induced by DEHP, flow cytometry analysis was performed using anti-PY labeled with CF647 dye (Biotium). As shown in Figure 4D, a significant increase in overall fluorescence was observed in DEHP treated cells resulting in 1.5- ± 0.2-fold increase in global fluorescence.

**FIGURE 5:**
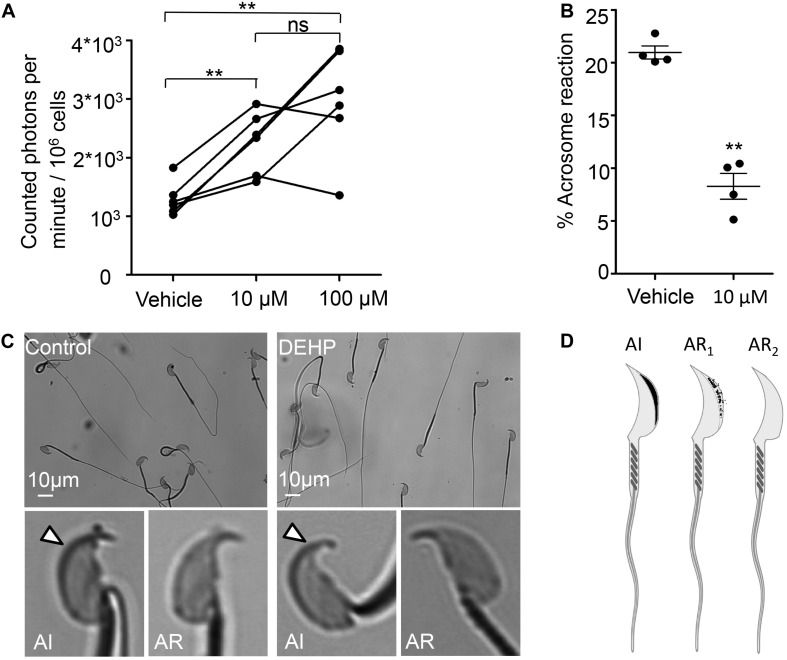
DEHP increases ROS production and decreases spontaneous acrosome reaction in capacitated sperm cells. **(A)** Luminol-dependent chemiluminescence assay showed increased rates of ROS production in sperm treated with 10 and 100 μM DEHP in comparison to the vehicle control (0 μM DEHP). Connected dots represent spermatozoa extracted from the same mouse (a total of six mice were used for this experiment). Each sample was divided into three aliquots and subjected to different conditions. A minimum of 1.35*10^6^ sperm cells/mL was used per condition. **(B)** Percentage of spontaneously acrosome-reacted spermatozoa subjected to 10 μM DEHP or vehicle control (0.1% ethanol) during capacitation. Each data point represents the mean of one of four independent experiments. A minimum of 500 cells was evaluated per experiment. **(C)** Acrosome-reacted (AR) and acrosome-intact (AI) spermatozoa in control and 10 μM DEHP. Arrowheads point at intact acrosome caps. “AI” cells have bright blue staining on the dorsal region of the acrosome. “AR” cells have patchy or absent staining. **(D)** Diagram showing acrosome-reacted (two distinct patterns are indicated as AR1 or partially reacted, and AR2 as fully reacted) and acrosome-intact (AI) spermatozoa. Images were produced using Biorender.com. Data are means ± S.E.M. ** indicates statistical significance (*P* < 0.01) between control spermatozoa and spermatozoa exposed to 10 μM DEHP.

The mid-piece region of sperm flagellum harbors mitochondria- an organelle known to generate ROS. Interestingly, DEHP increases ROS generation in various cells and tissues, including hepatocytes, adipocytes, and testis (Kasahara et al., 2002; Schaedlich et al., 2018; Huang et al., 2019). However, DEHP’s ability to alter sperm ROS production has not been studied. To detect ROS production, a chemiluminescence assay was employed, a commonly described technique to detect ROS in semen (Ochsendorf et al., 1994; Williams and Ford, 2005; Agarwal et al., 2008). The levels of ROS production were assessed in caudal sperm capacitated in the presence or absence of 10 and 100 μM DEHP after 60 min of exposure. A significant increase in ROS production was detected in all treated samples in comparison to vehicle-treated controls (Figure 5A). We found that already at 10 μM of DEHP, a maximal ROS production was achieved, and no further increase was detected at 100 μM. Since excessive ROS production is known to be cytotoxic to sperm, exposure to DEHP may lead to impaired sperm fertilizing capacity.

### The Acrosome Reaction in Capacitated Spermatozoa Is Inhibited by DEHP

The acrosome reaction is the fusion of the sperm plasma membrane with the outer acrosomal membrane. It is vital for fertilization, as only acrosome-reacted spermatozoa can fuse with the egg (Avella and Dean, 2011). According to previous reports, increased levels of ROS production result in excessive peroxidation of the sperm acrosomal membrane (Zalata et al., 2004), impairing acrosomal exocytosis and sperm-egg fusion (Aitken and Clarkson, 1987; Griveau et al., 1995; Ichikawa et al., 1999). While there is a debate over the physiological triggers for the acrosome reaction and the exact site of acrosomal exocytosis, it is well accepted that the acrosome reaction is required for sperm fertility. Therefore, we have explored whether DEHP can alter the spontaneous acrosome reaction. We found that incubation with 10 μM DEHP, decreased the percentage of spontaneous acrosome reaction. In control samples, the detected rates of acrosome reacted cells were 20.95 ± 0.62%, whereas in DEHP treated samples, the rates dropped to 8.28 ± 1.24% (Figures 5B–D).

To summarize, these results indicate that acute DEHP exposure stimulates excessive ROS production in sperm, as well as trigger altered tyrosine phosphorylation and inhibits the acrosome reaction. Consequently, these changes negatively affect sperm physiology and impact their fertility.

## Discussion

Exposure to EDCs poses a significant risk to reproductive health and fetal development (Hannon et al., 2016; Brehm et al., 2018). In this study, we assessed the effects of acute exposure to four omnipresent EDC’s – BPA, DEHP, DMP, and DEP on sperm fertility and early embryo development. While it is well documented that chronic exposure to these compounds can affect both male and female fertility (Latini et al., 2006; vom Saal et al., 2008; Kay et al., 2014; Wu et al., 2014; Patel et al., 2015), the effects of short exposure are less clear. To assess the impact of the chosen EDCs on sperm fertility and pre-implantation embryo development, we employed *in vitro* embryo development and IVF studies. Of all tested EDC’s DEHP had the most profound impact on fertilization and sperm fertility. While it is known that chronic exposure to DEHP impairs sperm motility and chromatin DNA stability (Pant et al., 2011; Sumner et al., 2019), its acute effect on sperm fertilizing capacity has not been elucidated. Here, we sought to investigate the impact of short exposure to DEHP on sperm fertility and the mechanisms by which this EDC exhorts its effects.

Upon absorption, DEHP is distributed throughout the body by the circulatory system. The majority of DEHP is quickly hydrolyzed by the liver into various metabolites, which have been linked to altered fertility and DNA damage in sperm (Hauser et al., 2007). However, a portion of DEHP is stored un-metabolized in the adipose tissue- which acts as a reservoir for lipophilic EDCs (Tanaka et al., 1975; Regnier and Sargis, 2014). Hormonal and neuronal signals regulating the fat tissue can trigger an abrupt release of lipophilic EDCs to the systemic circulation (Regnier and Sargis, 2014). There are two main ways in which sperm can encounter un-metabolized DEHP: via abrupt release from adipose tissue or through release from medical devices, such as a urological catheter- making unmetabolized DEHP a prominent threat to sperm fertility.

Di-2-ethylhexyl phthalate exposure has been previously linked to increased ROS production in somatic cells and oocytes (Ambruosi et al., 2011; Kim et al., 2013; Wu et al., 2014; Tripathi et al., 2019). However, its impact on ROS overproduction in sperm was not elucidated. Here we show that acute exposure to DEHP triggers excessive ROS generation, leading to oxidative damage and ultimately sperm infertility.

While minor ROS generation naturally takes place during early sperm capacitation, this process must be tightly regulated. Mild ROS production triggers an increase in intracellular cAMP, resulting in the activation of Protein Kinase A (PKA). PKA, in turn, carries out a series of controlled tyrosine phosphorylation events in a time-dependent manner (Aitken et al., 1995; Leclerc et al., 1997). Interestingly, certain EDCs such as BPA have been shown to up-regulate PKA’s activity leading to an altered phosphorylation pattern downstream of PKA (Rahman et al., 2015). Since mature spermatozoa are transcriptionally and translationally silent cells, post-translational protein modifications such as tyrosine phosphorylation play an essential role in sperm maturation process and their ability to fertilize an egg (Naz and Rajesh, 2004). Unwarranted ROS production leads to over-phosphorylation which significantly alters the maturation process of sperm (Villegas et al., 2003; Donà et al., 2011). Moreover, an excess of ROS is cytotoxic to sperm due to their limited antioxidant capacity and their high content of polyunsaturated long-chain fatty acids in the plasma membrane (Jones et al., 1979; Alvarez and Storey, 1982; Aitken and Clarkson, 1987).

Di-2-ethylhexyl phthalate-treated sperm cells had altered capacitation with aberrantly fast tyrosine phosphorylation within the first 60 min of capacitation. This differs from the gradual increasing protein phosphorylation pattern that was observed in the control condition and previously reported in the literature (Visconti et al., 1995b; Piehler et al., 2006). The detected increase in tyrosine phosphorylation was localized primarily to the mid-piece region of sperm, the flagellar compartment where mitochondria are located. Mitochondrial respiration produces a significant amount of ROS, this process, if unregulated, can damage sperm genomic DNA, lipid and protein structures, and subsequently impair sperm integrity and fertility. Previous reports show that DEHP exposed oocytes and somatic cells produce an excessive amount of ROS via mitochondrial-derived ROS (Rosado-Berrios et al., 2011; Wu et al., 2014; Roth, 2018). However, it has not been shown that DEHP affects spermatozoa in a similar manner. In fact, several EDCs were suggested to affect sperm fertility via CatSper-related mechanism (Schiffer et al., 2014). Here, we show that while murine CatSper was not sensitive to DEHP exposure, this phthalate indeed triggers excessive ROS production and subsequently impairs sperm fertility.

An additional effect of excessive oxidative stress on spermatozoa is lipid peroxidation. Spermatozoa are extremely susceptible to lipid peroxidation due to their high concentration of long- chain polyunsaturated fatty acids (Jones et al., 1979; Alvarez and Storey, 1982; Aitken and Clarkson, 1987; Aitken et al., 2006; Wathes et al., 2007). Alteration of the lipid structure due to peroxidation in sperm leads to a decrease in membrane fluidity (Sikka, 2001; Zalata et al., 2004; Cocuzza et al., 2007; Chen et al., 2013) causing decreased motility and altered acrosome reaction (Aitken and Clarkson, 1987; Griveau et al., 1995; Ichikawa et al., 1999; Zalata et al., 2004). The acrosome reaction is an important step during fertilization in order to expose sperm-egg recognition elements (Inoue et al., 2005). Murine sperm begin to undergo the acrosome reaction in the upper isthmus (La Spina et al., 2016), the part of the oviduct that connects the uterine with the ampulla. Thus, mouse fertile sperm are acrosome-reacted prior to reaching the ampulla, the site of fertilization and before encountering the eggs (Jin et al., 2011). In fact, most acrosome-intact spermatozoa are unable to fertilize the egg and swim away from the zona pellucida (Jin et al., 2011). Thus, sperm ability to undergo the AR at the end of capacitation is highly important for sperm fertility (Yanagimachi, 2011). Here we find that DEHP significantly inhibits the acrosome reaction in capacitated sperm. As a result, DEHP-exposed sperm were largely acrosome-intact and therefore unable to fertilize murine eggs. This explains the reduced levels of fertilization that were observed in IVF and the absence of pronuclei formation. These results indicate that, in addition to its chronic impact on reproductive potential, DEHP also imposes acute damage to sperm by affecting its ability to fertilize and thereby represent a risk to male fertility.

## Materials and Methods

### Animal Care

C57BL6 mice were purchased from Jackson Laboratory, Bar Harbor, ME, United States, or Harlan Laboratories (Indianapolis, IN, United States). The mice were kept in a room with controlled light (14 h light and 10 h darkness) and temperature (23 ± 0.5°C); 50–60% humidity. The mice were fed a standard chow diet (PicoLab Rodent diet 20 and LabDiet, 5053) and hyper-chlorinated water *ad libitum*. BPA, DEHP, DMP, and DEP (Sigma-Aldrich, St. Louis, MO, United States) were dissolved in ethanol (Sigma-Aldrich). Phthalates were used at a final concentration of 1, 2, and 10 μM. The concentration range of 1–10 μM for tested EDC’s was chosen based on previously reported phthalate concentrations linked to female infertility and on studies that evaluated DEHP plasma concentration in patients who undergo hemodialysis (Nassberger et al., 1987; Reddy et al., 2006). Vehicle controls were performed at the highest concentration.

### Embryo Collection From Natural Mating

Four- to-16- week-old female mice were super- ovulated by the standard procedure previously described (Hoogenkamp and Lewing, 1982). Briefly, 5 IU of pregnant mare serum gonadotropin (PMSG; EMD Millipore) were administered via intraperitoneal injection (i.p.) at 14:30. Forty-eight hours later, 5 IU of human chorionic gonadotropin (hCG; EMD Millipore) were injected. At the time of hCG injection, each female mouse was placed in an individual cage with one proven breeder (3–10 months old). The following morning, female mice were inspected for vaginal plugs. 20 h after hCG administration, embryos were dissected out from the oviducts. The isolated oocyte-cumulus complexes were placed in pre-warmed 50 μL droplet of Hyaluronidase (80 IU/mL) (LifeGlobal) and were gently pipetted up and down repeatedly in a fine glass pipette until the oocytes were partially denuded. The oocytes were then transferred to a pre-warmed M2 media (Zenith Biotech) supplemented with 4 mg/mL BSA (Sigma-Aldrich) and washed in 4–5 droplets until all the corona cells were removed. Zygotes from each individual mouse were randomly allocated to different culture conditions for 20 h of incubation. Since the tested EDCs show low water solubility and high oil solubility, the standard culture of embryos under oil could not be employed. Thus, we cultured the embryos in 500 μL KSOM (Zenith Biotech) ± EDCs at different concentrations for 20 h in 4-well dishes (*Nunc*^TM^, Sigma-Aldrich) without oil. At the end of the 20 h incubation, the two cell embryos were briefly washed and allocated to culture dishes, containing 10 μL droplets of KSOM (Zenith Biotech) supplemented with 1 mg/mL BSA (Sigma-Aldrich) overlaid with embryo-suitable light mineral oil (Millipore) in 5% CO_2_ and 37°C. Successful development was considered as morula or blastocyst – the final stage of embryonic development before implantation. To calculate the rate of embryo survival, we counted the number of embryos that developed to the morula or blastocyst stage and divided this number by the total number of the zygotes that were harvested.

### *In vitro* Fertilization

To investigate the influence of EDCs exposure on spermatozoa’s ability to fertilize eggs, *in vitro* fertilization (*IVF*) experiments were conducted. *IVF* was performed as previously described (Vergara et al., 1997) with a few modifications. Eggs were recovered from 4 to 16-week-old female mice by superovulation as described above. 13 h after hCG injection, the female mice were euthanized, and the oviducts were dissected out. The cumulus masses were isolated from the ampulla region of the oviduct, and incubated in HTF medium (Embryomax, Specialty Media, Millipore MR-070-D), 5% CO_2_, 37°C for 30 min prior to insemination. Sperm was obtained from a mature C57BL male mouse just before egg harvest. Spermatozoa were recovered by removing the caudae epididymis and placing each cauda separately in a petri dish containing pre-warmed HTF with or without EDCs. The tissue was cut five to six times, and sperm was allowed to swim out into the medium for 20–30 min. The cauda was then removed, and the resultant sperm suspension was left for an additional 30–60 min in the media at 37°C in 5% CO_2_ to capacitate. Total time of capacitation was 60–90 min.

Four well plates were used for fertilization. Each well was filled with 700 μL of HTF. Sperm was added to each well to a final concentration of 210,000 spermatozoa/mL. Subsequently, the cumulus masses were added to the fertilization dish; care was taken to avoid carry-over of excessive amounts of solution to maintain sperm concentrations. Since the cumulus masses were obtained around the time of ovulation, they were highly compact, making it difficult to quantify the exact number of eggs in each mass. To ensure similar numbers of eggs in each tested condition, two-three cumulus masses were added to each fertilization well. Dishes were placed in the incubator and maintained at 37°C in 5% CO_2_ for 4h. After that time, eggs were washed to remove excess sperm and then cultured in 10 μL droplets of KSOM supplemented with 1 mg/mL BSA and overlaid with embryo-tested light mineral oil in 5% CO_2_ and 37°C. To assess the rates of successful fertilization, the number of morula or blastocyst embryos produced by IVF on day 5 post insemination was counted and then divided by the number of eggs that were initially used for insemination.

### Electrophysiology

Sperm was collected as previously reported (Wennemuth et al., 2003). Giga-ohm seals between the patch pipette and mouse spermatozoa were formed at the cytoplasmic droplet. Seals were formed in HS solution comprising the following (in mM): 130 NaCl, 5 KCl, 1 MgSO_4_, 2 CaCl_2_, 5 glucose, 1 sodium pyruvate, 10 lactic acid, 20 HEPES, pH 7.4 adjusted with NaOH. Transition into the whole-cell mode was performed by applying short (1 ms) 499–611 mV voltage pulses, combined with light suction. Access resistance was 15–25 MΩ. Cells were stimulated every 5 s. Data were sampled at 2–5 kHz and filtered at 1 kHz. Pipettes (15–20 MΩ) for whole-cell patch-clamp recordings of monovalent CatSper currents were filled with the following (in mM): 130 Cs-methanesulfonate, 70 HEPES/MES, 3 EGTA, 2 EDTA, 0.5 Tris-HCl, pH 7.4 adjusted with CsOH. Bath divalent-free solution for recording of monovalent CatSper currents contained the following (in mM): 140 Cs-methanesulfonate, 40 HEPES/MES, 1 EDTA, pH 7.4 adjusted with CsOH. HS solution was used to record baseline current while measuring monovalent CatSper currents. 1 μL/mL EtOH (vehicle control), 10 μM or 100 μm DEHP were added to the bath solution right before electrophysiology experiments. CaCl_2_ was added to this solution in accordance with WinMAXC version 2.05 (C. Patton, Stanford University) to obtain the required free Ca^2+^ concentration.

### Capacitation of Spermatozoa in the Presence of 10 μM DEHP to Test the Level of Tyrosine Phosphorylation

Spermatozoa collection and the assessment of protein tyrosine phosphorylation was performed as previously outlined (Visconti et al., 1995a), with a few modifications. Spermatozoa were recovered by removing the cauda epididymis and placing it into a Petri dish containing HTF with either 1 μL/mL ethanol or 10 μM DEHP. The tissue was cut five to six times, and sperm was allowed to swim out for 15–20 min at 37°C in 5% CO_2_. The cauda was then removed, and spermatozoa suspension was further incubated at 37°C in 5% CO_2_. Sperm samples were collected after 30, 60, 90, and 120 min of capacitation and placed into a clean tube. After each sample collection, the sample was centrifuged at 21,000 × *g* for 1 min. The supernatant was discarded, and the cellular pellet was resuspended in 25 μL of 2× Laemmli sample buffer (Bio-Rad) (Laemmli, 1970). The sample was then boiled for 5 min at 95°C and centrifuged at 21,000 × *g* for 1 min. Supernatants were transferred to clean tubes, β-mercapto-ethanol was added (to a final concentration of 2.5%), and the sample was heated again to 95°C for 1 min. 20 μL of the total crude cell lysate from each sample was loaded onto a 4–20% gradient Tris-HCl Criterion SDS-PAGE (Bio-Rad). After transfer to polyvinylidene fluoride membrane, blots were blocked in 0.1% PBS-Tween20 (Fisher Scientific) with 3% IgG-free BSA blocking solution for 1 h and incubated with anti-phosphotyrosine antibody, clone 4G10 (Millipore, 05-321) at a dilution of 1:2000 in 1% IgG-free BSA blocking solution overnight at +4°C. The membrane was then washed three times in PBST and probed with a secondary horseradish peroxidase-conjugated antibody (Abcam) at a dilution of 1:15,000 in 1× PBST. After subsequent washing, the membrane was developed with an ECL SuperSignal West Pico kit (Pierce) according to the manufacturer’s instructions. After detection, the membrane was stripped and re-probed with mouse tubulin-alpha ab-2 (Sigma-Aldrich), 1:5000 dilution. To quantify the global changes in tyrosine phosphorylation, rectangular boxes were drawn around each lane of the western blots’ images. Each lane’s optical density was normalized. First, the detected signals were normalized to the loading control, acetylated tubulin. Subsequently, each lane was normalized to the control lane at 120 min of capacitation.

### Spontaneous Acrosome Reaction in the Presence of 10 μM DEHP in Capacitated Spermatozoa

Assessment of the acrosomal status was done as previously reported (Larson and Miller, 1999) with a few adjustments. In summary, the right and left caudae epididymides were surgically removed. One cauda was placed in HTF medium supplemented with 1 μl/mL ethanol while the second one was placed in HTF medium containing 10 μM DEHP. Each cauda was cut five to six times, and sperm were allowed to swim out for 15–20 min at 37°C in 5% CO_2_. The cauda was then removed, and the resultant spermatozoa suspension was left to capacitate for 60 min. On average, the concentration of sperm in each condition was 2–5 × 10 cells/mL. After 60 min of capacitation, spermatozoa suspension was transferred to clean microtubes and centrifuged at 300 × *g* for 5 min at room temperature. The cells were then fixed in 4% PFA in 1X PBS for 15 min. At the end of fixation, an equal volume of 0.1 M ammonium acetate was added. The microfuges were centrifuged at 800 × *g* for 5 min. The supernatant was removed, and sperm cells were resuspended in the remaining 100 μL. 30 μL of sperm suspension was spotted onto non-charged microscope slides and gently spread out with a glass pipette. The samples were allowed to air-dry for 15 min. Subsequently, the slides were washed in Milli-Q water followed by a methanol wash, and then Milli-Q water again, each wash step was done for 5 min. The slides were subsequently submerged in Coomassie brilliant blue (Sigma-Aldrich) solution for 2 min (0.11 g Coomassie brilliant blue, 20 mL water, 25 mL Methanol, and 5 mL glacial acetic acid). Next, the slides were rinsed in Milli-Q water to remove excess Coomassie and mounted with Mowiol mounting medium (Millipore). After the cells were mounted, the acrosomal status was immediately assessed to avoid diffusion of the stain. Acrosome intact cells had bright blue staining on the dorsal region of the acrosome. Acrosome reacted cells had patchy or absent staining. 500 sperm cells per condition (100–200 cells per slide, 3–5 slides per condition) were assessed.

### Flow Cytometry

Spermatozoa was capacitated in the presence of either 10 μM DEHP or vehicle control. To detect phosphotyrosine residues in capacitated sperm by flow cytometry we followed the methodology previously outlined (Barbonetti et al., 2008), with few modifications. Sperm aliquots (3 × 10^6^) were taken at 60 min of capacitation and fixed in 3.7% PFA in 1 × PBS for 10 min at room temperature. To remove the PFA, the cells were centrifuged at 500 × *g* for 10 min. The supernatant was removed, and the cellular pellet was resuspended in 1 × PBS. The cells were washed twice. Next, the cells were permeabilized in 0.1% Triton X-100 for 10 min at RT. Non-specific binding sites were blocked by 0.1% BSA in PBST for 30 min at RT. To detect phosphotyrosine, we conjugated an anti-phosphotyrosine antibody clone 4G10 with a CF 647 dye (Mix-n-Stain^TM^ Antibody Labeling Kit, Biotium), as per the manufacturer’s instructions. To label the cells, sperm were incubated with 10 μg/ml of the conjugated antibody in PBS with 0.1% BSA for 1 h at RT. Labeled spermatozoa were then washed in PBS and resuspended in 250 μL PBS for flow cytometric analysis. 10,000 cells per sample were analyzed. Sperm fluorescence was quantified using the BD LSR Fortessa flow cytometer equipped with an argon laser tuned far red spectrum. flow cytometry analysis was performed with the aid of a positive and a negative control for each experiment. FlowJo^TM^ Software was used for data analysis. The region of interest was selected based on sperm forward scatter (FSC, relative cell size) and side scatter (SSC, cell internal complexity) to eliminate cellular debris.

### ROS Production Detection by Chemiluminescence Assay

Spermatozoa were recovered by removing both caudae epididymides and placing them into a 30 mm Petri dish containing HS media. Cells were allowed to swim out for 15–20 min. Subsequently, the sperm suspension was equally divided between three Eppendorf tubes and spun down at 300 × *g* for 7 min. After the removal of the supernatant, the cells were resuspended in an equal volume of HTF containing either ethanol or DEHP. The control tube contained 1 μL/mL ethanol and the treatment tube contained either 10 μM or a 100 μM DEHP. The suspensions were then capacitated at 37°C in 5% CO_2_ for 60 min. Detection of reactive oxygen species generated by sperm cells was done using the chemiluminescent agent – luminol following a previously described procedure (Saleh and Agarwal, 2002; Agarwal et al., 2008). The chemiluminescent probe, luminol (Sigma-Aldrich, A8511-5G.) was freshly prepared before each experiment. After 60 min of capacitation, the samples were spun down at 300 × *g* for 7 min and resuspended in 125 μM luminol in DPBS. Negative control, test sample, and positive control were prepared. 100 μL of 30% hydrogen peroxide solution was added to the positive control. A 100 μL aliquot of the cell suspension was taken from each sample for sperm count. The samples were then taken for Chemiluminescence measurements using the Lumicycle 32 (Actimetrics, Inc., Wilmette, IL, United States). The luminometer measured Chemiluminescence at 37°C for 5 min. ROS production was expressed as counted photons per minute (CPM)/10^6^ sperm. Data were recorded using Actimetrics Lumicycle Data Collection software and analyzed using the Actimetrics Lumicycle Analysis program.

### Statistical Analyses

For statistical analyses used in the manuscript the GraphPad Prism 5 software (GraphPad Software, Inc., La Jolla, CA, United States) was used. Unpaired *t*-test was used to determine statistical significance for embryo survival, *IVF* and Chemiluminescence experiments, and assigning *p* ≤ 0.05 as the limit. Paired *t*-test was used for the AR, PY and flow cytometry experiments. All results are shown with the standard error of the mean. The significance of changes are indicated as follows: ^∗^*p* ≤ 0.05, ^∗∗^*p* ≤ 0.01, ^∗∗∗^*p* ≤ 0.001.

## Data Availability Statement

All datasets generated for this study are included in the article/Supplementary Material.

## Ethics Statement

All experiments were performed in accordance with NIH guidelines for animal research and approved by UC Berkeley Animal Care and Use Committee (AUP 2015-07-7742), with every effort made to minimize suffering for the animals.

## Author Contributions

LGK and PL conceived the project, designed the experiments, and wrote the manuscript. LGK performed all studies, data acquisition, and analysis for the manuscript. JD helped with acrosome reaction, tyrosine phosphorylation, chemiluminescence, and flow cytometry studies. NP assisted with flow cytometry experiments. JM and LJK helped with lumicycle and data analysis of luminol studies. All authors discussed the results and commented on the manuscript.

## Conflict of Interest

The authors declare that the research was conducted in the absence of any commercial or financial relationships that could be construed as a potential conflict of interest.

## Supplementary Material

FIGURE S1 Murine embryo survival post exposure to 2 μM of tested EDCs. *In vitro* embryo development on day 5 post fertilization. **(A)** Representative images of blastocysts previously exposed at the zygote stage to 2 μM DEP for 20 h. **(B)** Representative images of blastocysts previously exposed at the zygote stage to 2 μM DMP for 20 h. Scale bars are 50 μm.

FIGURE S2 Fertilization rate of murine eggs exposed to spermatozoa treated with 2 μM of tested EDCs. **(A)** Representative images of blastocysts obtained after murine eggs were introduced to sperm previously exposed 2 μM DEP. **(B)** Representative images of blastocysts obtained after murine eggs were introduced to sperm previously exposed 2 μM DMP. **(C)** Representative images of blastocysts obtained after murine eggs were introduced to sperm previously exposed 2 μM BPA. Scale bars are 50 μm.

FIGURE S3 Murine CatSper is not affected by DEHP. **(A)** Representative monovalent whole-cell CatSper currents (*I*_*CatSper*_) recorded from a murine spermatozoon in the absence (black) and presence of 10 μM (blue) and 100 μM DEHP (red). *I*_*CatSper*_ were activated by a voltage ramp from −80 to +80 mV from a holding potential of 0 mV. Voltage protocol is shown above the currents. The panel on the right shows the main conducting ion of the pipette and bath solutions. **(B)** Averaged *I*_*CatSper*_ densities recorded from murine epididymal spermatozoa in the absence and presence of DEHP. Data are means ± S.E.M. An average of 3 independent experiments is shown.

FIGURE S4 Representative dot plot of side (SSC-A) versus forward (FSC-A) scatter showing flow-cytometry data obtained for sperm. **(A)** The region of interest demarcated by solid lines was selected to eliminate cellular debris. **(B–F)** Representative flow cytometry histograms from five independent experiments. Mean fluorescence intensities (MFI) normalized to mode show an increase in global tyrosine phosphorylation in 10 μM DEHP treated spermatozoa (red) at 60 min of capacitation compared to the vehicle control (blue). The background fluorescence detected in unstained spermatozoa is shown in gray. Tyrosine phosphoproteins were detected using a CF 647 dye conjugated to anti-PY (monoclonal antibody).

FIGURE S5 Time course of capacitation-associated tyrosine phosphorylation of specific sperm proteins is altered by exposure to DEHP. Levels of relative tyrosine phosphorylation obtained from each of the four protein bands at the corresponding molecular weights: 75, 95, 170, and 270 kDa. **(A)** The density of the 75 kDa protein band was normalized to the densities of the loading control, followed by normalization to the control at 120 min. Each data point represents the average of one of the three independent experiments. **(B)** The 95 kDa protein band normalized as in **(A)**. **(C)** The 170 kDa protein band normalized as in **(A)**. **(D)** The 270 kDa protein band normalized as in **(A)**. Normalization to the control band at 120 min was chosen as the strongest physiological phosphorylation signal.

TABLE S1 **(A–D)** Murine embryo development after 20 h exposure to DMP, BPA, DEP, or DEHP. Embryo development was assessed on day 5 post fertilization. The column “progression to blastocyst stage per experiment, %” represents the percentage of embryos that reached blastocyst or morula stage. This number was calculated by dividing the number of all embryos that reached blastocyst or morula stage to the number of all collected zygotes per each experiment. Zygotes were obtained from naturally mated super-ovulated females. Each condition was assessed by 3–8 independent experiments. **(A)** Embryo development after 20 h exposure to DMP at 0, 1, 2, and 10 μM and subsequent embryo culture in DMP-free media. **(B)** Embryo development after 20 h exposure to the indicated concentration of BPA and subsequent culture in BPA-free media. **(C)** Embryo development after 20 h exposure to the indicated concentration of DEP and subsequent culture in DEP-free media. **(D)** Embryo development after 20 h exposure to the indicated concentration of DEHP and subsequent culture in DEHP-free media.

TABLE S2 The development of murine zygotes isolated from naturally mated super-ovulated females and after their exposure to 0.1% ethanol for 20 h in the culture media. Embryo development was assessed on day 5 post fertilization and represents the percentage of embryos that reached blastocyst or morula stage. This number was calculated by dividing the number of all embryos that reached blastocyst or morula stage by the number of all collected zygotes per experiment.

TABLE S3 **(A–D)** Development of *in vitro* fertilized mouse embryos obtained after murine eggs were introduced to the sperm previously exposed to DMP, BPA, DEP, or DEHP for 60–90 min. Embryo development was assessed on day 5 post fertilization. The “Progression to the blastocyst stage per experiment, %” column represents the percentage of embryos that reached blastocyst or morula stage. This number was calculated by dividing the number of all embryos that reached blastocyst or morula stage to the number of all collected and inseminated eggs per each experiment. Each condition was assessed by 3–5 independent experiments. **(A)** *In vitro* embryo development after eggs insemination with 0, 1, 2, or 10 μM DMP-treated sperm **(B)** *In vitro* embryo development after eggs were inseminated with spermatozoa previously exposed to the indicated concentration of BPA. **(C)** *In vitro* embryo development after eggs were inseminated with the spermatozoa treated with corresponding concentrations of DEP. **(D)** *In vitro* embryo development after murine eggs were inseminated with spermatozoa treated with corresponding concentrations of DEHP.

TABLE S4 Development of embryos derived from the murine eggs that were subjected to *in vitro* fertilization (IVF) with murine sperm previously exposed to 0.1% ethanol for 60–90 min. Embryo development was assessed on the day 5 post IVF and represents the percentage of embryos that reached blastocyst or morula stage. This number was calculated by dividing the number of all embryos that reached blastocyst or morula stage to the number of all collected eggs per each independent experiment.

TABLE S5 Effect of 10 μM DEHP on fertilization and pronuclei formation. Pronucleus (PN) formation was assessed 9 h after egg insemination with sperm cells previously exposed to 10 μM DEHP. Three independent experiments were carried out.

TABLE S6 Assessment of the global tyrosine phosphorylation by flow cytometry. Tyrosine phosphoproteins were detected by flow cytometry using a CF 647 dye conjugated to anti-PY antibody. Five independent experiments were carried out. Sperm concentrations were normalized between all conditions for each experiment. Values are mean fluorescence intensity (MFI) detected in the APC-Cy7-A channel normalized to mode. A ratio of the normalized detected MFI in the 10 μM DEHP-treated sperm cells vs. the vehicle control was used to calculate the fold increase in global fluorescence.

TABLE S7 Detection of reactive oxygen species (ROS) in murine spermatozoa treated with DEHP compared with vehicle control. The luminol-dependent chemiluminescence assay was used to detect ROS production. Six independent experiments were carried out. A minimum of 1.35^∗^10^6^ sperm cells/mL were used per condition. ROS production was expressed as counted photons per minute (CPM)/10^6^. Each row represents an individual experiment. For each experiment, sperm concentrations were normalized between all conditions.
