# Supplementary material for: The Impact of Di-2-Ethylhexyl Phthalate on Sperm Fertility
Source: Front Cell Dev Biol. 2020 Jun 30;8:426. doi: 10.3389/fcell.2020.00426 (PMC7338605; doi:10.3389/fcell.2020.00426)
Supplement: FIGURE S1 — Murine embryo survival post exposure to 2 μM of tested EDCs. In vitro embryo development on day 5 post fertilization. (A) Representative images of blastocysts previously exposed at the zygote stage to 2 μM DEP for 20 h. (B) Representative images of blastocysts previously exposed at the zygote stage to 2 μM DMP for 20 h. Scale bars are 50 μm. [file Data_Sheet_1.PDF]

Supplemental Figure 1

20h exposure

A 2μM DEP

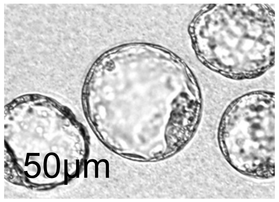

B 2μM DMP

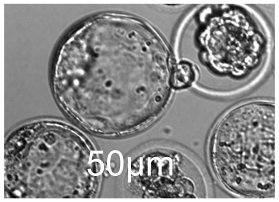

Supplemental Figure 2

In vitro fertilization

A 2µM DEP

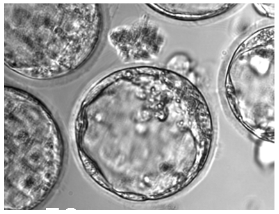

B 2µM DMP

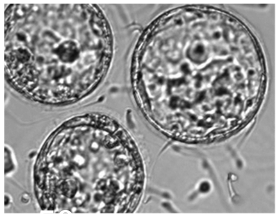

C 2µM BPA

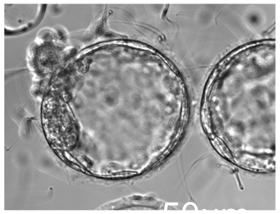

Supplemental Figure 3

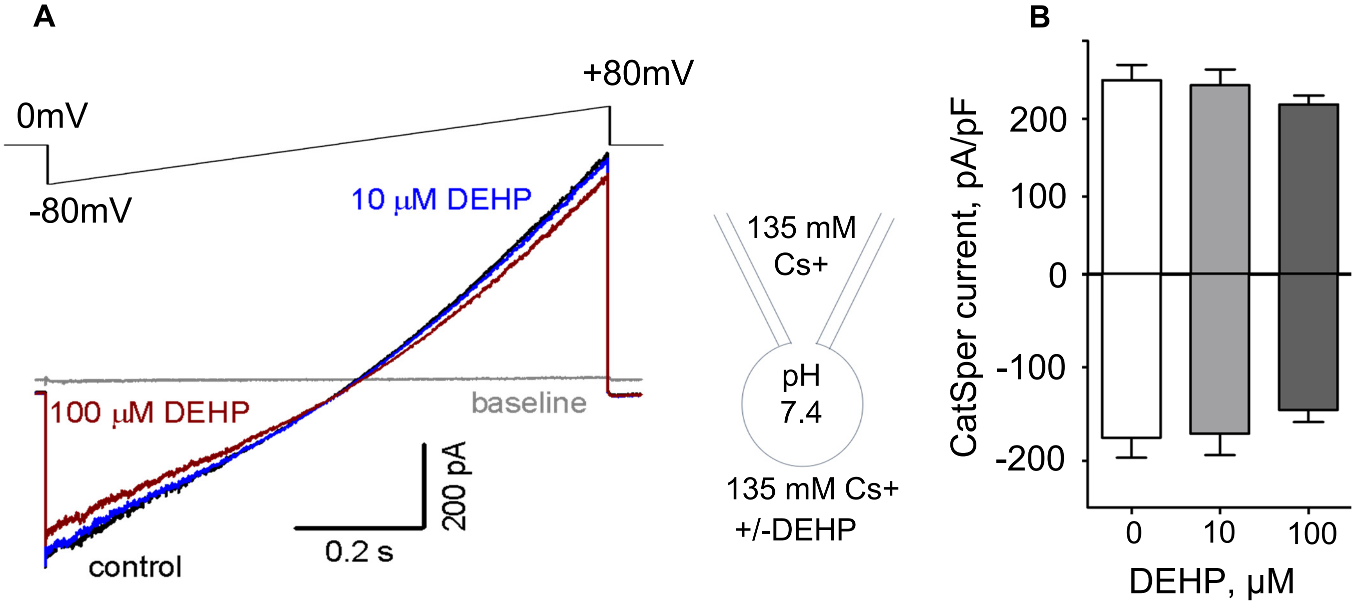

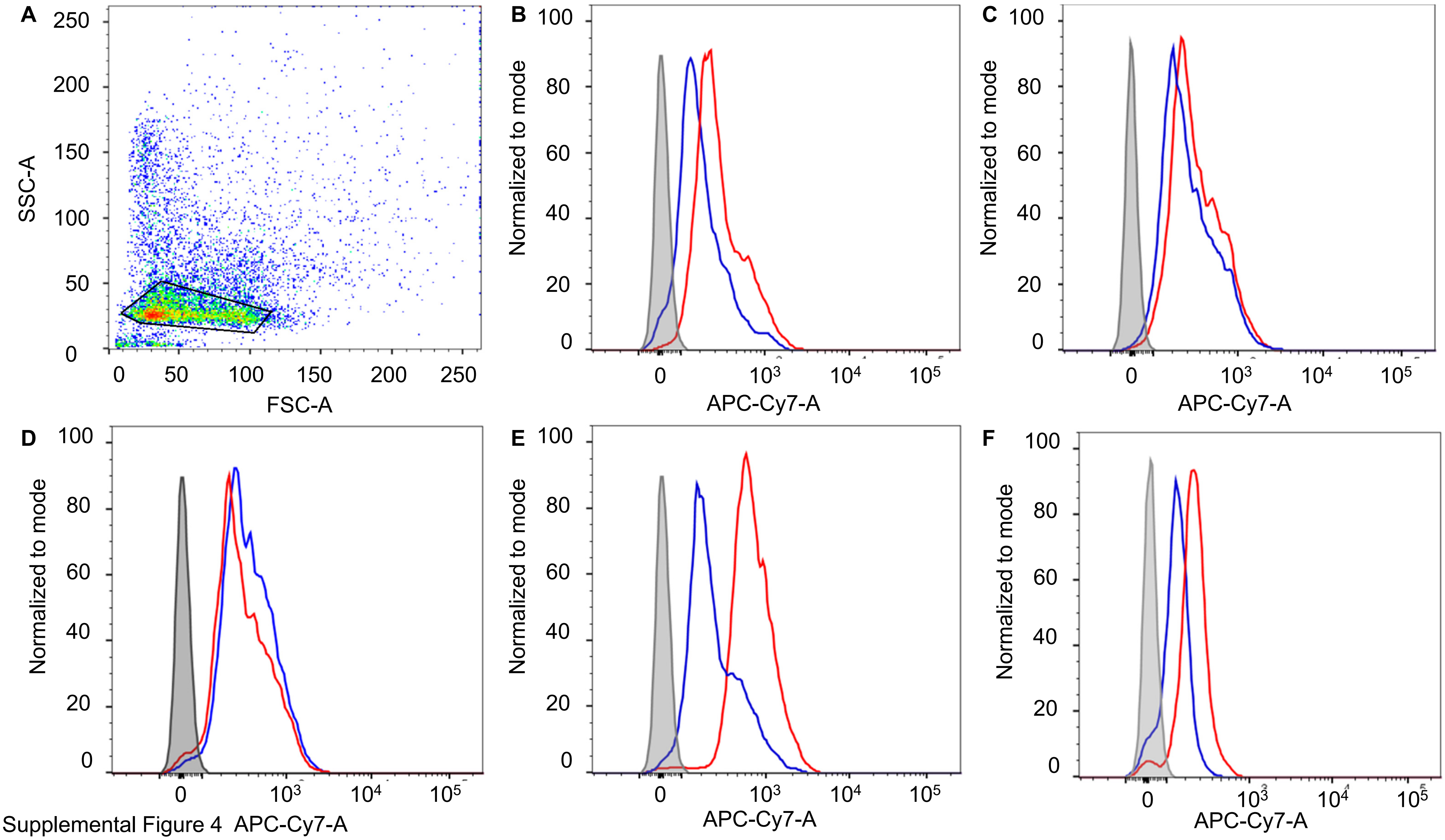

Supplemental Figure 4

Supplemental Figure 5

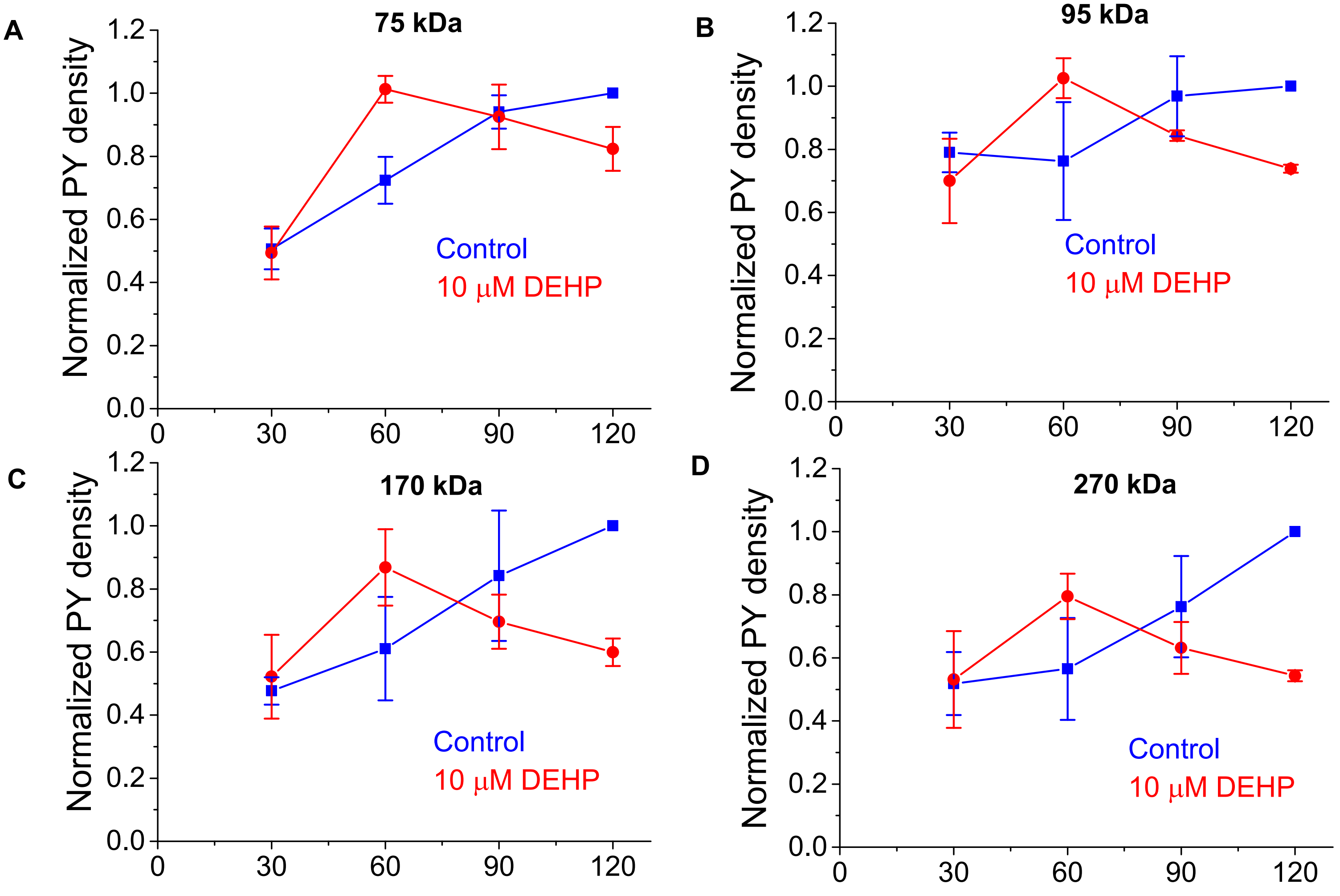

**Table 1A** 20h exposure to DMP	Number of independent trials	Progression to blastocyst stage per experiment,%	Number of blastocysts/number of zygotes collected
Control	1	100%	37/38
	2	83%	
	3	100%	
	4	100%	
	5	100%	
1μM DMP	1	61%	26/46
	2	80%	
	3	33%	
	4	40%	
	5	100%	
2μM DMP	1	41%	14/34
	2	100%	
	3	57%	
	4	0%	
10μM DMP	1	0%	0/31
	2	0%	
	3	0%	
	4	0%	

**Table 1B** 20h exposure to BPA	Number of independent trials	Progression to blastocyst stage per experiment,%	Number of blastocysts/number of zygotes collected
Control	1	50%	35/42
	2	83%	
	3	82%	
	4	100%	
	5	100%	
1μM BPA	1	91.6%	25/27
	2	100%	
	3	90%	
2μM BPA	1	33.3%	13/20
	2	66.7%	
	3	87.5%	
10μM BPA	1	0%	0/37
	2	0%	
	3	0%	

**Table 1C** 20h exposure to DEP	Number of independent trials	Progression to blastocyst stage per experiment, %	Number of blastocysts/number of zygotes collected
Control	1	100%	27/29
	2	100%	
	3	85%	
	4	100%	
1μM DEP	1	68.75%	34/41
	2	100%	
	3	85.70%	
	4	92.80%	
2μM DEP	1	100%	21/34
	2	71.50%	
	3	0%	
	4	92%	
10μM DEP	1	0%	0/33
	2	0%	
	3	0%	

**Table 1D** 20h exposure to DEHP	Number of independent trials	Progression to blastocyst stage per experiment,%	Number of blastocysts/number of zygotes collected
Control	1	90%	44/48
	2	85.70%	
	3	100%	
	4	100%	
	5	100%	
	6	83%	
1μM DEHP	1	30%	13/23
	2	75%	
	3	100%	
2μM DEHP	1	100%	41/51
	2	66.7%	
	3	85%	
	4	75%	
	5	100%	
	6	0%	
	7	90%	
	8	100%	
10μM DEHP	experiments 1 to 4	0%, 0%, 0%, 0%	0/56

Table 2. Vehicle control 1μL/mL 20h exposure

20h exposure to Vehicle Control,1μL/mL	Number of independent trials	Progression to blastocyst stage per experiment,%	Number of blastocysts/number of zygotes collected
Vehicle Control,1μL/mL	1	100%	16/17
	2	83%	
	3	100%	

**Table 3A** IVF; DMP	Number of independent trials	Progression to blastocyst stage per experiment,%	Number of blastocysts/number of eggs collected
Control	1	71.4%	26/36
	2	71%	
	3	80%	
	4	67%	
1μM DMP	1	48%	30/42
	2	89.5%	
	3	83%	
2μM DMP	1	69%	28/36
	2	100%	
	3	87.5%	
	4	67%	
10μM DMP	1	57%	21/30
	2	43%	
	3	82%	
	4	100%	

**Table 3 B** IVF; BPA	Number of independent trials	Progression to blastocyst stage per experiment,%	Number of blastocysts/number of eggs collected
Control	1	100%	24/28
	2	88%	
	3	77%	
	4	50%	
1μM BPA	1	62%	56/88
	2	73%	
	3	63%	
	4	63%	
2μM BPA	1	90%	39/47
	2	88%	
	3	67%	
10μM BPA	1	73%	13/19
	2	63%	
	3	48%	

**Table 3C** IVF; DEP	Number of independent trials	Progression to the blastocysts stage per experiment, %	Number of blastocysts/number of eggs collected
Control	1	90%	28/31
	2	94.7%	
	3	50%	
1µM DEP	1	88.8%	20/28
	2	82%	
	3	38%	
2µM DEP	1	89%	35/42
	2	78%	
	3	70%	
10µM DEP	1	80%	18/20
	2	100%	
	3	50%	

**Table 3D** IVF; DEHP	Number of independent trials	Progression to the blastocyst stage per experiment,%	Number of blastocysts/number of eggs collected
Control	1	86%	72/92
	2	87%	
	3	89%	
	4	88%	
	5	77%	
	6	65%	
	7	47%	
	8	67%	
1µM DEHP	1	77%	16/18
	2	100%	
	3	100%	
2µM DEHP	1	58%	25/51
	2	60%	
	3	13%	
	4	66%	
	5	42%	
10µM DEHP	experiments 1 to 7	0%, 0%, 0%, 0%, 0%, 0%, 0%, 7%	1/63

Table 4. Vehicle control 1μL/mL IVF

20h exposure to Vehicle Control,1μL/mL	Number of independent trials	Progression to blastocyst stage per experiment,%	Number of blastocysts/number of zygotes collected
	1	100%	28/37
	2	80%	
	3	100%	
	4	50%	

**Table 5** **PN formation**	Number of independent trials	Progression to blastocyst stage per experiment,%	Number of blastocysts/number of zygotes collected
Control	1	84.6%	21/25
	2	71.4%	
	3	60%	
DEHP 10μM	1	0%	2/36
	2	0%	
	3	10%	

**Table 6** **Flow cytometry** Mean Fluorescence Intensity (MFI) APC-Cy7-A normalized to mode	Number of independent trials	Control MFI	DEHP MFI	MFI Ratio DEHP/Control
	1	151	260	1.72
	2	374	442	1.18
	3	265	402	1.52
	4	523	514	0.98
	5	358	859	2.39

Table 7: Luminometer counts per minute (CPM)/ 10^6 sperm

Mouse #	cell/mL in vehicle	Vehicle CPM	CPM/ 10^6 sperm vehicle	cell/mL in 10µM	10µM CPM	CPM/ 10^6 sperm 10µM	cell/mL in 100µM	100µM CPM	CPM/ 10^6 sperm 100µM
1	$2.57*10^6$	4694	1826.459	$2.17*10^6$	6321.666	2913.210	$2.525*10^6$	6750.333	2673.399
2	$1.4*10^6$	1520	1085.714	$1.375*10^6$	3214	2337.454	$1.4*10^6$	5345.333	3818.095
3	$1.425*10^6$	1777.333	1247.251	$1.9*10^6$	3216	1692.631	$2.775*10^6$	3769	1358.198
4	$1.35*10^6$	1612	1194.074	$2.1*10^6$	3329.666	1585.555	$1.775*10^6$	5127.666	2888.826
5	$2.375*10^6$	3232	1360.842	$2.25*10^6$	5983.666	2659.407	$1.675*10^6$	5278	3151.044
6	$1.575*10^6$	1619.666	1028.359	$1.475*10^6$	3526.333	2390.734	$1.225*10^6$	4722.333	3854.965
